# Efficacy and safety of daily home-based transcranial direct current stimulation as adjunct treatment for bipolar depressive episodes: Double-blind sham-controlled randomized clinical trial

**DOI:** 10.3389/fpsyt.2022.969199

**Published:** 2022-09-20

**Authors:** Jangwon Lee, Chan Woo Lee, Yoonjeong Jang, Ji Seon You, Yun Seong Park, Eunjeong Ji, Hyeona Yu, Sunghee Oh, Hyun A. Ryoo, Nayoung Cho, Ji Yoon Park, Joohyun Yoon, Ji Hyun Baek, Hye Youn Park, Tae Hyon Ha, Woojae Myung

**Affiliations:** ^1^Department of Neuropsychiatry, Seoul National University Bundang Hospital, Seoul, South Korea; ^2^Medical Research Collaborating Centre, Seoul National University Bundang Hospital, Seoul, South Korea; ^3^Department of Psychiatry, Samsung Medical Centre, Sungkyunkwan University School of Medicine, Seoul, South Korea; ^4^Department of Psychiatry, Seoul National University College of Medicine, Seoul, South Korea

**Keywords:** bipolar depressive episodes, transcranial direct current stimulation, clinical trial, double-blind, efficacy, safety

## Abstract

**Background:**

Although transcranial direct current stimulation (tDCS) is known to be a promising therapeutic modality for unipolar depression, the efficacy and safety of tDCS for bipolar depressive episodes (BD) are still unknown and clinical trials of home-based tDCS treatment are scarce. As a result, we set out to investigate the efficacy and safety of home-based tDCS for the treatment BD.

**Methods:**

Participants (*n* = 64), diagnosed as bipolar disorder as per the diagnostic and statistical manual of mental disorders (DSM-5), were randomly assigned to receive tDCS. Hamilton Depression Rating Scale (HDRS-17) scores were measured at the baseline, week 2, 4, and 6, and home-based tDCS (for 30 min with 2 mA) was self-administered daily.

**Results:**

Of the 64 patients (15.6% bipolar disorder I, 84.4% bipolar disorder II), 41 patients completed the entire assessment. In the intention-to-treat analysis, time-group interaction for the HDRS-17 [*F*_(3, 146.36)_ = 2.060; *p* = 0.108] and adverse effect differences between two groups were not statistically significant, except the pain score, which was higher in the active group than the sham group (week 0–2: *p* < 0.01, week 2–4: *p* < 0.05, and week 4–6: *p* < 0.01).

**Conclusion:**

Even though we found no evidence for the efficacy of home-based tDCS for patients with BD, this tool was found to be a safe and tolerable treatment modality for BD.

**Clinical trial registration:**

[https://clinicaltrials.gov/show/NCT03974815], identifier [NCT03974815].

## Introduction

Bipolar disorder is a chronic and severe mental illness (1), In bipolar disorder depressive episodes are more chronic and more common than (hypo) manic episodes (2). Pharmacological treatments are standard for bipolar depressive episodes (BD), but they have limitations such as inadequate efficacy and common adverse effects (AE) that include sedation, weight gain, and teratogenicity (3). Moreover, few pharmacological treatments have proven to be highly and consistently effective in BD (4). Due to these limitations there is increasing interest in non-pharmacological approaches that encompass cognitive-behavioral therapy, psychoeducation, family-focused interventions, and neuromodulation. Transcranial direct current stimulation (tDCS) is a non-invasive form of treatment that involves application of a very low amplitude (1–3 mA) direct electrical current to the scalp (3) and an alternative therapeutic option to other neuromodulation modalities such as electroconvulsive therapy (ECT) or repetitive transcranial magnetic stimulation (rTMS). Given the necessity of hospitalization and anesthesia associated with ECT, the high cost, and risk of seizures associated with rTMS (5) tDCS is more tolerable for patients (6). Furthermore, the frequency of AEs seems to be low for tDCS (7).

Recent meta-analyses (8, 9) have suggested that tDCS is effective for treating unipolar depression. Moreover, double-blind randomized clinical trials (RCTs) (10, 11) and a meta-analysis (12) have reported that tDCS is an effective and safe augmentation option for BD. These results show the benefits of tDCS in the context of BD. However, in real-world situations, the traditional tDCS setting has drawbacks such as requiring daily visits to the hospital, transportation costs, disruption of daily activities, and work schedules, which decrease patient compliance (13). Thus, home-based tDCS was designed to address these limitations (14) and no critical side effects have been revealed to date (15). However, research on the efficacy and safety of home-based tDCS in patients with BD is lacking.

Therefore, we aimed to examine the efficacy and safety of home-based tDCS for treating BD. We conducted a randomized sham-controlled double-blind clinical trial involving participants in a home-based setting for 6 weeks. As evaluated by changes in the 17-item Hamilton Depression Rating Scale (HDRS-17) scores after 6 weeks of treatment, we hypothesized that active tDCS would have larger antidepressant effects than sham tDCS. We also hypothesized that tDCS would significantly alleviate depression symptoms, as defined by other efficacy measures, and that AE rates would be comparable in both groups.

## Patients and methods

The study was conducted at the Seoul National University Bundang Hospital, from July 2019 to May 2021. The study used a parallel design in which 64 patients were randomly assigned, by a computer-generated list using random block sizes, to the sham or the active tDCS group. The research protocol was approved by the Seoul National University Bundang Hospital Institutional Review Board and registered with ClinicalTrials.gov (NCT03974815, https://clinicaltrials.gov/show/NCT03974815). Written informed consent was obtained from all the participants. The study followed the ethical principles of the Declaration of Helsinki.

### Participants

Participants were recruited by physician referrals and in-hospital poster advertisements. They were pre-screened via in-person interviews, and those who met the general criteria were subjected to additional screening. All participants were screened by trained, board-certified psychiatrists who used the modified Mini-International Neuropsychiatric Interview (16) to diagnose BP (type I or II) in a major depressive episode.

To be included in the study, patients had to be between 19 and 65 years of age and have active symptoms of a current depressive episode [4 or more on the Clinical Global Impression Severity of Bipolar Scale (CGI−BP)] (17). We included patients taking mood stabilizers (lithium, divalproex, or lamotrigine) for at least 4 weeks before the day of screening. We considered the first-, second-, or third-line pharmacotherapies for adequate pharmacologic intervention in accordance with the Canadian Network for Mood and Anxiety Treatments (CANMAT) 2018 bipolar guidelines (18). Quetiapine, lithium, lamotrigine, valproate sodium, aripiprazole adjuvant therapy, carbamazepine, and venlafaxine adjuvant therapy were considered valid for bipolar I and II depressive episodes. In addition to patients treated with the CANMAT first-, second-, or third-line pharmacotherapies, those treated with propranolol, gabapentin, olanzapine, quetiapine, risperidone, ziprasidone, amisulpride, aripiprazole, clozapine, or benzodiazepines were also included according to their AE profile or symptomatology.

The study excluded individuals with a history of neurological disease, intellectual disability, cognitive impairment (inability to understand instructions or operate equipment), or those with a high risk of suicide that required hospitalization. Those who had metal equipment, coils, and electronic devices (such as cochlear implants or heart pacemakers) were also excluded. We also excluded those who had dermatological problems, such as an allergic skin reaction on the location of the electrodes. Women of childbearing potential who did not agree to use the medically permitted methods of contraception (such as barrier contraceptives, oral contraceptives lasting for at least 3 months, injection or insertion contraceptives, intrauterine contraceptives) for up to 24 weeks after using the tDCS medical device were also excluded.

Patient losses occurred if (1) they did not visit the hospital at weeks 2, 4, or 6; (2) compliance was less than 60%; (3) during the trial, there were serious clinical or psychiatric problems, such as suicide attempt/ideation or full-blown manic or hypomanic episodes; (4) patients were excluded for safety reasons, such as a significant worsening of their psychiatric condition or serious AEs; or (5) they withdrew their participation voluntarily.

### Intervention

Patients used a tDCS stimulation device at home and were trained to use it sufficiently by the research personnel. The tDCS procedure was performed using MINDD STIM (Ybrain Inc., Seoul, South Korea). This equipment can provide information about and when the patient applies the device and for how long. Patients were instructed to use the device within 2 h of setting it up. The anode and cathode were used for delivering electrical stimulation. Patients attached 28.26 cm^2^ round electrodes, a montage known as the “bifrontal” setup (F3-Anode, F4-Cathode) that had been previously used in a major depression trial (19). The anode and cathode electrodes were placed over the left and right DLPFC, respectively. All patients read the instructions with the researcher and watched videos related to how to use the tDCS so that they could learn how to use the tDCS. In addition, when the first stimulation was performed at the hospital, the researcher confirmed with the patient whether they could use the device correctly on their own. When patients had any questions about how to use the device, they were able to contact the researcher, and they resolved their difficulties in using the device through voice or video calls. All patients were retrained on how to use it at each visit, and they were able to ask any questions they had about how to use the tDCS.

For up to 42 sessions, tDCS was applied for 30 min daily. For the verum tDCS condition, a constant current of 2 mA was delivered for 29 min with additional ramp-up and ramp-down phases of 30 s each at the beginning and the end of the session, respectively. For the sham tDCS condition after 30 s of ramp-up and 30 s of ramp-down, the device was turned off. Patients were asked to report any discomfort, including adverse events, on the list provided at enrollment. In addition, every time the patient visited the hospital, we inquired about any tDCS-related discomfort and pain during the tDCS usage by using Numeric Rating Scale. Neither the researcher nor the patient knew which stimulus-set tDCS they received until the end of the study to prevent researcher expectancy response bias. The appearance of the tDCS was the same, but the stimuli for each group were different, and neither the researcher nor the patient knew the difference.

Using the information recorded in the smartphone application connected to the tDCS, the researchers were able to confirm whether the participants completed the 30-min sessions. When participants were in an environment with no smartphone internet connection, a diary was created and confirmed.

### Outcomes

All the evaluations were carried out by the blinded researchers. Participants were evaluated at baseline, week 2, week 4, and week 6 of the study. At baseline, and weeks 2, 4, and 6, adverse events were documented. The primary outcome was the change in the HDRS-17 (20) score between groups over time. The secondary outcomes included (1) changes in the Hamilton Anxiety Rating Scale (HAM-A) (21), Young Mania Rating Scale (YMRS) (22), CGI-BP (17), and Quality of Life Enjoyment and Satisfaction Questionnaire—Short Form (QLESQ-SF); (2, 23) the AE rates. The summary of intervention and measurement periods are presented in Supplementary Table 1.

### Statistical analyses

All statistical analyses were performed using R, version 4.0.5 (lme4 package; R Foundation, Vienna, Austria). The sample size was estimated at a power of 80% and a two-tailed α level of 5%. Based on a previous study evaluating the efficacy of tDCS in BD (10) we obtained a sample size of 56 participants. Assuming an attrition rate of 15%, we obtained a total sample size of 64 participants. We performed an intention-to-treat analysis. Differences in the baseline clinical and demographic variables of the groups were analyzed using *t*-tests or χ^2^-tests for continuous and categorical variables, respectively. The Wilcoxon rank sum test was also used for non-parametric data. The primary outcome was analyzed with a linear mixed-effect model incorporating group (two levels: active and sham) and time (four levels: baseline, weeks 2, 4, and 6), as well as their interaction, as independent variables, and the participant as a random-effects variable. The HDRS-17 score served as the dependent variable in the model. We also performed subgroup analyses based on bipolar type (I or II), sex, age (<40 or ≥ 40), HDRS-17 score (≤median or > median), HAM-A score (≤median or > median), and medication usage (lithium, valproic acid). The potential confounding variables, including age, sex, diagnosis (bipolar I or II), current episode duration, and baseline HDRS-17 score were adjusted. Considering the regularity of the test intervals (2 weeks), an autoregressive covariance structure was assumed as the working correlation matrix. The main hypothesis was that there would be a significant interaction between time and group, with active tDCS outperforming the sham over time. The frequencies of AEs at weeks 2, 4, and 6 were compared between the groups using Fisher’s exact test.

## Results

### Participants

**FIGURE 1:**
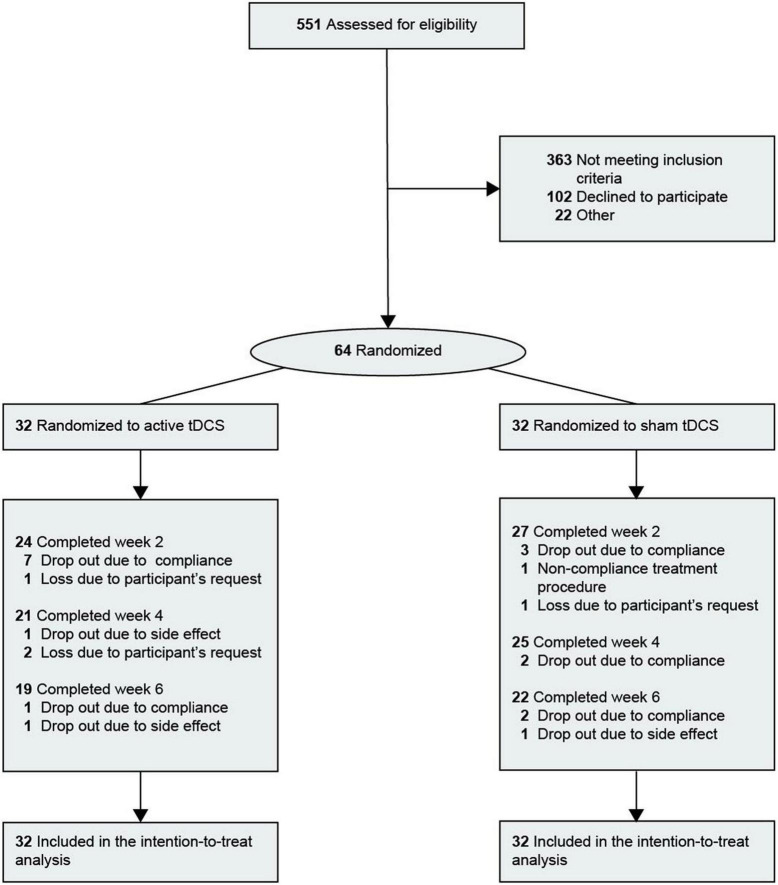
Flow diagram of participant selection. Ten participants were lost in the sham group and 12 participants were lost in the active group. tDCS, transcranial direct current stimulation.

The clinical and demographic characteristics of the patients are presented in Table 1. Of the 64 patients, 47 (73.4%) were women. The mean [standard deviation (SD)] age was 33.4 years (12.6 years). The proportion of patients with bipolar I disorder was 15.6%. The study flow chart is shown in Figure 1. Of the 64 patients included, 41 (19 in the active group and 22 in the sham group) received full 30-min tDCS sessions ranging from 18 to 42 days (mean = 38.00, SD = 4.69) and completed the final assessment. Thirteen patients were lost in the active group (8 under 60% compliance, 3 withdrawals, 1 due to participant’s request, and 2 due to AE) and 10 patients were lost in the sham group (7 under 60% compliance, 1 non-compliance with the treatment procedure, 1 withdrawal due to participant’s request, and 1 adverse effect).

**TABLE 1:** Clinical and demographic characteristics of the study sample at baseline.

Characteristic	No. (%)		
	
	Sham (*n* = 32)	Active (*n* = 32)	Total (*n* = 64)
**Demographics**			
Women	24 (75.0)	23 (71.9)	47 (73.4)
Age, mean (*SD*), years	31.16 (11.9)	35.66 (13.1)	33.41 (12.6)
Years at school, mean (*SD*)	13.72 (2.3)	14.16 (2.5)	13.94 (2.4)
Employed	11 (34.4)	16 (50.0)	27 (42.2)
Married	6 (18.8)	9 (28.1)	15 (23.4)
BMI, mean (*SD*)	23.83 (3.2)	24.00 (3.6)	23.91 (3.4)
**Clinical characteristics**			
Onset age, mean (*SD*), y	19.69 (8.8)	22.00 (7.6)	20.84 (8.2)
**Bipolar disorder**			
Type I	5 (15.6)	5 (15.6)	10 (15.6)
Type II	27 (84.4)	27 (84.4)	54 (84.4)
Previous episodes, mean (*SD*), No.	12.50 (11.7)	12.69 (14.3)	12.59 (12.9)
Current episode duration > 12 months	9 (28.1)	13 (40.6)	22 (34.4)
Severe depression	27 (84.4)	29 (90.6)	56 (87.5)
Generalized anxiety disorder	21 (65.6)	16 (50.0)	37 (57.8)
Panic disorder	17 (53.1)	16 (50.0)	33 (51.6)
Social anxiety disorder	7 (21.9)	7 (21.9)	14 (21.9)
Any anxiety disorder	4 (12.5)	2 (6.3)	6 (9.4)
**Pharmacotherapies in the present episode**			
First-line treatments being used, mean (SD), no.^†^	2.03 (0.7)	1.84 (0.8)	1.94 (0.7)
**Antidepressant drugs**			
SSRIs	4 (12.5)	3 (9.4)	7 (10.9)
Venlafaxine	1 (3.1)	1 (3.1)	2 (3.1)
Bupropion	2 (6.3)	0	2 (3.1)
**Mood stabilizers^‡^**			
Lithium	29 (90.6)	26 (81.3)	55 (85.9)
Valproate	12 (37.5)	12 (37.5)	24 (37.5)
Lamotrigine	17 (53.1)	14 (43.8)	31 (48.4)
Carbamazepine^§^	0	1 (3.1)	1 (1.6)
**Antipsychotics**			
Quetiapine	19 (59.4)	19 (59.4)	38 (59.4)
Olanzapine	4 (12.5)	3 (9.4)	7 (10.9)
Clozapine	5 (15.6)	3 (9.4)	8 (12.5)
Aripiprazole	11 (34.4)	10 (31.3)	21 (32.8)
Risperidone	6 (18.8)	2 (6.3)	8 (12.5)
Other SGAs^‡⁣‡^	2 (6.3)	10 (31.3)	12 (18.8)
**Other treatments**			
Benzodiazepines^¶^	8 (25.0)	3 (9.4)	11 (17.2)
Other anticonvulsants^†⁣†^	6 (18.8)	4 (12.5)	10 (15.6)

BMI, body mass index (calculated as weight in kilograms divided by height in meters squared); No., number; SD, standard deviation; SGAs, second-generation antipsychotics; SSRIs, selective serotonin reuptake inhibitors. ^†^First-line treatment for bipolar depressive episode per 2018 Canadian Network for Mood and Anxiety Treatments (CANMAT) guidelines. ^‡^Recommended for bipolar depression treatment. ^§^ Third-line treatment for bipolar depressive episode per 2018 CANMAT guidelines. ^¶^ Clonazepam, lorazepam, alprazolam, and etizolam. ^†⁣†^Gabapentin and topiramate. ^‡⁣‡^Ziprasidone and amisulpride.

### Integrity of blinding

There was no significant difference between the active and sham groups in relation to the likelihood of correctly guessing to which group they were assigned to (χ^2^ = 0.065, *p* = 1.000, 54.8 and 51.6% in the active and sham groups, respectively). Therefore, we assumed that the participants were unable to infer their actual groupings.

### Primary and secondary outcomes

**FIGURE 2:**
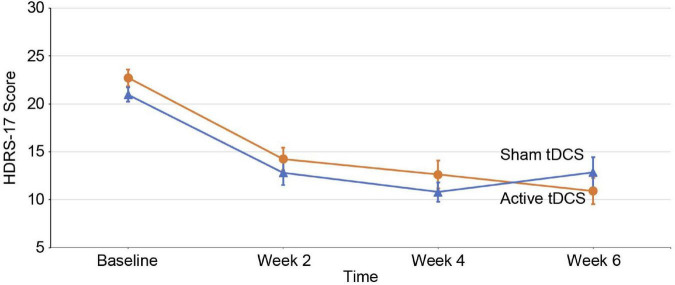
Changes in depression scores over time. Mean changes in 17-item Hamilton Depression Rating Scale (HDRS-17) scores (intention-to-treat analysis) from baseline to endpoint. Error bars indicate 1 standard error. The X-axis represents the hospital visit date and the Y-axis represents the HDRS-17 score evaluated for that week. tDCS, transcranial direct current stimulation.

The primary outcome was changes in the HDRS-17 scores. The linear mixed model analysis showed that there was no significant time-group interaction for the HDRS-17 score [*F*_(3, 146.36)_ = 2.060; *p* = 0.108] (Figure 2). We also did not find a significant time-group interaction for the HDRS-17 score in the subgroup analysis (Supplementary Table 2). There were no significant time-group interactions between the other secondary outcomes (Supplementary Table 3).

### The frequencies of adverse effects in the groups were compared

The frequencies of all AEs were not significantly different. There were no treatment-emergent affective switch (TEAS) episodes during the trial (Table 2). We also examined suicidal ideation, aggressive behavior, and elated mood as side effects. Four participants reported suicidal ideation during weeks 2–4 and 4–6, and one participant reported an episode of aggressive behavior during weeks 4–6. The frequency of these events did not differ for the active and sham groups (*p* > 0.99 for suicidal ideation at weeks 2–4 and 4–6; *p* = 0.47 for aggressive behavior at weeks 4–6). The pain score was significantly higher in the active group than in the sham group during weeks 0–2 (*p* < 0.01), 2–4 (*p* < 0.05), and 4–6 weeks (*p* < 0.01).

**TABLE 2:** Frequency of adverse events and mean score of pain and discomfort^**†**^.

Adverse event	Weeks 0–2			Weeks 2–4			Weeks 4–6		
			
	No. (%)		*P*-value^‡^	No. (%)		*P*-value^‡^	No. (%)		*P*-value^‡^
					
	Sham (*n* = 27)	Active (*n* = 24)		Sham (*n* = 25)	Active (*n* = 24)		Sham (*n* = 23)	Active (*n* = 20)	
Headache	1 (3.7)	0 (0.0)	>0.99	−	−	−	−	−	−
Neck pain	−	−	−	−	−	−	−	−	−
Tingling	2 (7.4)	2 (8.3)	>0.99	1 (4.0)	4 (16.7)	0.19	2 (8.7)	4 (20.0)	0.39
Itching	−	−	−	−	−	−	−	−	−
Burning	−	−	−	0 (0.0)	1 (4.2)	0.49	−	−	−
Skin redness	0 (0.0)	2 (8.3)	0.22	−	−	−	−	−	−
Sleepiness	−	−	−	−	−	−	−	−	−
Trouble concentrating	−	−	−	−	−	−	−	−	−
Fatigue	−	−	−	−	−	−	−	−	−
Nausea	−	−	−	−	−	−	−	−	−
Dizziness	−	−	–	−	−	−	0 (0.0)	1 (5.0)	0.47
Suicidal ideation	−	−	−	1 (4.0)	1 (4.2)	>0.99	1 (4.3)	1 (5.0)	>0.99
Aggressive behavior	−	−	−	−	−	−	0 (0.0)	1 (5.0)	0.47
Skin color	−	−	−	−	−	−	0 (0.0)	1 (5.0)	0.47
Elated mood	−	−	−	−	−	−	−	−	−
TEAS episode	−	−	−	−	−	−	−	−	−
Pain (10-point Likert scale) and discomfort score (4-point Likert scale)									

	**Sham (*n* = 27)**	**Active (*n* = 24)**	***P*-value^‡^**	**Sham (*n* = 25)**	**Active (*n* = 23)**	***P*-value^‡^**	**Sham (*n* = 22)**	**Active (*n* = 20)**	***P*-value^‡^**

Pain score, mean (*SD*)	1.39 (1.1)	2.38 (1.2)	0.004	1.17 (1.0)	1.97 (1.5)	0.03	0.96 (0.7)	1.96 (1.4)	0.006
Discomfort score, mean (*SD*)	0.85 (0.5)	0.92 (0.3)	0.58	0.79 (0.4)	0.83 (0.4)	0.76	0.72 (0.5)	0.79 (0.4)	0.64

NA, not applicable; TEAS, treatment-emergent affective switch. ^†^Adverse events were assessed using an adverse effects questionnaire. During transcranial direct current stimulation (tDCS) application, all participants were asked to complete this questionnaire daily, describing the presence of an adverse event. ^‡^P-values were determined using χ^2^ or Fisher’s exact test and independent t-test or Wilcoxon rank-sum test. One sham-allocated participant did self-harm and concluded not related to tDCS application. Two active-allocated participants who reported suicidal ideation discontinued the further study.

## Discussion

In this study, we found that the active tDCS group did not show symptomatic improvement superior to that of the sham tDCS group. The frequency of AE did not differ in the active tDCS and sham groups. Tolerability was consistent with previous studies (10, 24, 25). Those who received tDCS reported significantly higher pain scores than the sham group during weeks 0–2, 2–4, and 4–6. However, the average pain score of the active tDCS group was 2.10 on a 10-point Likert scale, which indicated tolerability.

Our findings for efficacy were in contrast with those of previous double-blind RCT studies (10).

A previous study reported that active tDCS had superior symptomatic improvement than sham tDCS, based on the HDRS-17 scores. Two other studies that used small-sized open-label designs and involved patients with unipolar and BD reported that tDCS is efficacious for depressive symptoms (26, 27). One possible explanation for the lack of efficacy in our study could be the different inclusion criteria that allowed for the concurrent use of medication. In our study, all patients were on at least one mood stabilizer during the study period. Mood stabilizers such as lithium, valproic acid, and lamotrigine can modulate cortical excitability, the mechanism associated with voltage-gated sodium channels (28, 29). Lithium selectively inhibits the function of voltage-gated sodium channels (30), and anticonvulsant-based mood stabilizers also target voltage-gated sodium channels (31, 32). This blockade of voltage-gated sodium leads to reduced neuronal excitability (33). Reduced cortical excitability may be associated with a poorer antidepressant response for tDCS (34–36). Another possible explanation for the discrepancy could be the significantly lower usage of antidepressant medications than in previous studies (10, 26, 27). No participants in our study were receiving antidepressant monotherapy. In addition, only a few patients were prescribed antidepressants along with mood stabilizers (17.2%). Nitsche et al. (37) reported that enhancing serotonergic activity increases and prolongs facilitatory plasticity and converts the inhibitory plasticity into facilitation. Thus, enhancing the serotonergic activity of antidepressants may enhance the plasticity effect of tDCS (37), which may affect the efficacy of tDCS. In clinical practice, most guidelines (18, 38, 39) recommend combination therapy with mood stabilizers or antipsychotics rather than antidepressant monotherapy for BD. Therefore, our study design reflects the clinical guidelines for BD. However, our study did not directly compare the antidepressant use and non-use groups; therefore, further research is needed. A third possible explanation for the lack of efficacy in our study could be the variable response to the sham condition. A single-blind parallel tDCS study (40) for healthy participations suggested that sham conditions previously assumed to be inactive may alter neuronal function. A systematic review (41) of tDCS for depression showed that the sham response in tDCS depression trials was large. The possibility that the sham conditions of 30 s of ramp-up and 30 s of ramp-down may have had a stimulation effect cannot be excluded.

The tolerability results of tDCS in our study were consistent with those of other studies (10, 24, 25), that reported no significant difference in AEs between the active and sham groups. Another systematic review (42) of tDCS did not have conclusions for tolerability, not because of AEs, but most involved studies did not report adequate AEs. Four patients reported suicidal ideation. The number of occurrences was equal in active and sham groups. There was no occurrence of TEAS, including manic switching or suicidal attempt, which is a concern when treating BD (43–45). This finding is similar to the outcome of previous studies (10, 26, 27) even though our study participants received more frequent stimulation [up to 42 times compared to 30 of previous studies (10, 26, 27)]. These AE findings postulate the tolerability of tDCS for BD.

Similar to the primary outcome results, the active tDCS group did not show a significant difference from the sham group for the secondary outcomes. Although previous double-blind RCTs (10) reported no significant difference in CGI-BP scores between the active and sham groups, few studies have reported on YMRS, Q-LES, and HAM-A for BD. A randomized controlled study (46) reported no improvement in the HAM-A scores in patients with generalized anxiety disorder using tDCS. No significant difference in the YMRS scores between the active and sham groups seems to support the safety of tDCS for BD related to manic switch (47). We performed subgroup analysis and found no significant results within each group. However, these results should be interpreted with caution due to the limited sample size.

There have been three home-based tDCS studies, one for major depressive disorder (48), chronic stroke (49), and Alzheimer’s dementia (50), but none have examined tDCS for BD. In our study, 8 participants in the active group (25.0%) and 7 participants in the sham group (21.9%) dropped out due to low compliance. The dropout rate in our study was higher than previous studies on chronic stroke (49) and Alzheimer dementia (50). One major difference is that caregivers applied tDCS in these previous studies, whereas in our study, patients applied tDCS on themselves. Moreover, the requirement to apply the device over the weekend might have also decreased the compliance in our study. Comparing previous RCT studies for tDCS in patients with mood disorder (10, 26, 27), the dropout rate in our study was comparable. These results suggests that home-based tDCS is feasible and applicable in outpatient and home-based settings.

This is the first study of home-based tDCS for BD patients, and it suggests the safety and tolerability of tDCS for BD, even with a relatively high number of stimulations. Furthermore, we monitored compliance not only by self-report but also with smartphone data, which improved the reliability of the compliance rate. In addition, this study was a double-blind RCT, and the integrity of blinding was adequate.

This study had several limitations. First, the fact that each participant was on different medications could have affected the efficacy of tDCS. Allowing various medications such as mood stabilizers, benzodiazepines, antipsychotics, and antidepressants may have made it difficult to examine the efficacy of tDCS. Future clinical trials that restrict medication variance and medication subgroup analyses are required. Second, this study was conducted in single-center with a limited sample size. There is a need for large samples and multicenter home-based clinical trials to investigate the efficacy of home-based tDCS. The study also unexpectedly revealed a dropout rate of 25% in the active group due to restrictions on distance movement restrictions implemented during the COVID-19 pandemic. Although there were high drop out rates and no positive association between tDCS and efficacy in our sample size, our study results suggested the directions of future studies as an exploratory study. Third, the sham condition designed to maintain the integrity of blinding had minimal electrical stimulation, so this may have affected the efficacy analysis. It may be necessary to develop a sham condition without electrical stimulation while maintaining the integrity of blinding. In addition, we verified the patients’ compliance but could not confirm whether the patient used the device correctly at home, which is a major weakness of home-based device designed study. In order to compensate for this limitation, this study allowed enough time for device education in order for patients to use the tDCS by themselves through researcher’s demonstration and watching videos of handling the device. This device education was focused on how the patients could correctly position the electrodes, and this training sessions were held multiple times on the patients’ visit whenever it was necessary. Further studies will still need to check patients’ compliance, but will need sophisticated systems to check whether patients use the tDCS as the suggested directions.

## Conclusion

tDCS was not proven effective but was found to be both tolerable and safe in this home-based trial conducted for patients with bipolar I or II disorders. The negative results of our study should be re-examined in further studies with larger samples.

## Data availability statement

The datasets generated during and/or analyzed during the current study are available from the corresponding author upon reasonable request.

## Ethics statement

The studies involving human participants were reviewed and approved by the Institutional Review Board, Seoul National University Bundang Hospital (E-1902-523-002). The patients/participants provided their written informed consent to participate in this study.

## Author contributions

WM and TH had full access to all the data in the study and took responsibility for the integrity of the data, the accuracy of the data analysis, contributed to concept and design, critical revision of the manuscript for important intellectual content, and supervision. CL, YJ, YP, and HY were involved in data acquisition. JL, CL, YJ, and JSY drafted the manuscript. CL and EJ contributed to statistical analysis. WM obtained the funding. All authors who met authorship criteria were listed as authors, and certify that they participated sufficiently in the work to take public responsibility for the content, including participation in the concept, design, analysis, writing, or revision of the manuscript and contributed to interpretation of data and writing of the manuscript.
