# Supplementary material for: Efficacy and safety of daily home-based transcranial direct current stimulation as adjunct treatment for bipolar depressive episodes: Double-blind sham-controlled randomized clinical trial
Source: Front Psychiatry. 2022 Sep 20;13:969199. doi: 10.3389/fpsyt.2022.969199 (PMC9530445; doi:10.3389/fpsyt.2022.969199)
Supplement: Supplementary file 1 [file Data_Sheet_1.doc]

**Supplementary Table S1. Summary of intervention and measurement periods**

|  | **VISIT 1** | | **Treatment** | **VISIT 2** | **Treatment** | **VISIT 3** | **Treatment** | **VISIT 4** |
| --- | --- | --- | --- | --- | --- | --- | --- | --- |
|  | **Screening** | **Baseline** | **Home** | **F/U** | **Home** | **F/U** | **Home** | **F/U** |
|  |  |  |  | **2 Week** |  | **4 Week** |  | **6 Week** |
| **Consent form** | ● |  |  |  |  |  |  |  |
| **Inclusion and exclusion criteria** | ● | |  |  |  |  |  |  |
| **MINI** | ● | |  |  |  |  |  |  |
| **Education of using tDCS equipment** |  | ● |  | ● |  | ● |  |  |
| **Applying tDCS** |  | ● | ● |  | ● |  | ● |  |
| **HDRS-17** | ● | |  | ● |  | ● |  | ● |
| **HAM-A** | ● | |  | ● |  | ● |  | ● |
| **Q-LES** | ● | |  | ● |  | ● |  | ● |
| **YMRS** | ● | |  | ● |  | ● |  | ● |
| **CGI** | ● | |  | ● |  | ● |  | ● |
| **Adverse effects questionnaire** |  | ● |  | ● |  | ● |  | ● |

**Abbreviations:** F/U, Follow-up, MINI, Mini international neuropsychiatric interview. HDRS-17, 17-item Hamilton Depression Rating Scale. HAM-A, Hamilton Anxiety Rating Scale. Q-LES, Quality of Life Enjoyment and Satisfaction. YMRS, Young Mania Rating Scale. CGI, Clinical Global Impression scale.

**Supplementary Table S2. Results of linear mixed models on HDRS-17 score according to subgroup**

| **Subgroup** | **N (sham/real)** | **Sum of Squares** | **Mean Square** | ***F-*Value** | ***P-*Value** |
| --- | --- | --- | --- | --- | --- |
| Bipolar disorder |  |  |  |  |  |
| Type I | 5 / 5 | 19.39 | 6.46 | 0.27 | 0.85 |
| Type II | 27 / 27 | 88.10 | 29.37 | 1.83 | 0.14 |
| Age |  |  |  |  |  |
| < 40 | 24 / 19 | 49.09 | 16.36 | 1.02 | 0.39 |
| ≥ 40 | 8 / 13 | 69.44 | 23.15 | 1.17 | 0.33 |
| Gender |  |  |  |  |  |
| Male | 8 / 9 | 15.40 | 5.13 | 0.38 | 0.77 |
| Female | 24 / 23 | 121.16 | 40.39 | 2.25 | 0.09 |
| Lithium |  |  |  |  |  |
| Being used | 29 / 26 | 83.85 | 27.95 | 1.64 | 0.18 |
| Unused | 3 / 6 | 106.74 | 35.58 | 2.20 | 0.14 |
| Valproic Acid |  |  |  |  |  |
| Being used | 12 / 12 | 162.59 | 54.20 | 2.74 | 0.05 |
| Unused | 20 / 20 | 8.21 | 2.74 | 0.18 | 0.91 |
| HDRS-17 |  |  |  |  |  |
| ≤ median | 19 / 16 | 5.94 | 1.98 | 0.15 | 0.93 |
| > median | 13 / 16 | 129.86 | 43.29 | 2.23 | 0.09 |
| HAM-A |  |  |  |  |  |
| ≤ median | 19 / 13 | 28.41 | 9.47 | 0.69 | 0.56 |
| > median | 13 / 19 | 191.58 | 63.86 | 3.29 | 0.03 |

*Note*, Interactive effects on HDRS-17 score are shown according to Subgroup, Time (baseline, weeks 2, 4, and 6) × Group (sham vs active).

**Supplementary Table S3. Clinical Measurements over Time**

|  | **Group** | **Baseline** | **Week 2** | **Week 4** | **Week 6** | **Interaction**  (Time × Group) |
| --- | --- | --- | --- | --- | --- | --- |
| **Intention to treat, mean ± SD** | | | | | | **p-value^1^** |
| **HDRS-17** | Active | 22.72±4.89 | 14.25±5.74 | 12.62±7.00 | 10.90±6.06 | 0.19 |
|  | Sham | 20.97±4.25 | 12.81±6.45 | 10.80±4.84 | 12.86±7.38 |  |
| **HAM-A** | Active | 20.97±9.23 | 16.75±7.95 | 15.24±9.77 | 14.85±9.42 | 0.67 |
|  | Sham | 18.78±9.34 | 13.70±8.90 | 12.56±8.41 | 14.00±9.67 |  |
| **Q-LES** | Active | 41.06±6.78 | 43.54±9.13 | 42.14±9.52 | 44.45±11.27 | 0.41 |
|  | Sham | 37.22±6.98 | 41.30±8.58 | 42.20±7.21 | 40.64±6.53 |  |
| **YMRS** | Active | 3.19±2.01 | 3.33±2.73 | 2.81±2.79 | 3.35±4.76 | 0.86 |
|  | Sham | 3.34±2.38 | 3.78±4.15 | 3.00±2.61 | 2.82±3.26 |  |
| **CGI** | Active | 4.22±0.42 | 3.46±1.06 | 3.14±0.91 | 3.10±0.97 | 0.66 |
|  | Sham | 4.22±0.42 | 3.74±1.06 | 3.44±1.00 | 3.32±0.95 |  |
| **Per protocol, mean ± SD** | | | | | |  |
| **HDRS-17** | Active | 23.15±5.51 | 14.50±6.12 | 12.75±7.15 | 10.90±6.06 | 0.15 |
|  | Sham | 21.45±4.78 | 13.23±6.87 | 10.91±5.15 | 12.86±7.38 |  |
| **HAM-A** | Active | 22.95±10.00 | 17.50±8.29 | 15.40±10.00 | 14.85±9.42 | 0.39 |
|  | Sham | 18.14±9.58 | 15.00±9.22 | 12.91±8.93 | 14.00±9.67 |  |
| **Q-LES** | Active | 38.00±6.24 | 42.75±9.57 | 42.45±9.65 | 44.45±11.27 | 0.30 |
|  | Sham | 37.32±8.13 | 40.68±8.14 | 42.77±7.36 | 40.64±6.53 |  |
| **YMRS** | Active | 3.35±2.11 | 3.15±2.62 | 2.85±2.85 | 3.35±4.76 | 0.87 |
|  | Sham | 3.23±2.47 | 3.50±3.65 | 2.82±2.67 | 2.82±3.26 |  |
| **CGI** | Active | 4.20±0.41 | 3.30±0.80 | 3.15±0.93 | 3.10±0.97 | 0.60 |
|  | Sham | 4.27±0.46 | 3.73±1.16 | 3.41±1.05 | 3.32±0.95 |  |

Abbreviations: HDRS-17, 17-item Hamilton Depression Rating Scale. HAM-A, Hamilton Anxiety Rating Scale. Q-LES, Quality of Life Enjoyment and Satisfaction. YMRS, Young Mania Rating Scale. CGI, Clinical Global Impression Scale.

For the per protocol scores, mean (SD) values were estimated based in the complete sample.

Greenhouse-Geisser approach was used if sphericity was not assumed.

^1^ *p*-values are for interactive effect (Time × Group).
